# KRAS Mutation-Responsive miR-139-5p inhibits Colorectal Cancer Progression and is repressed by Wnt Signaling

**DOI:** 10.7150/thno.45971

**Published:** 2020-06-05

**Authors:** Feng Du, Tianyu Cao, Huahong Xie, Ting Li, Lina Sun, Hao Liu, Hao Guo, Xin Wang, Qi Liu, Taewan Kim, Jeffrey L Franklin, Ramona Graves-Deal, Weili Han, Zuhong Tian, Minghui Ge, Yongzhan Nie, Daiming Fan, Robert J Coffey, Yuanyuan Lu, Xiaodi Zhao

**Affiliations:** 1 State Key Laboratory of Cancer Biology and National Clinical Research Center for Digestive Diseases, Xijing Hospital of Digestive Diseases, Fourth Military Medical University, Xi'an, Shaanxi 710032, China.; 2 Department of Internal Medicine, The Hospital of the People's Liberation Army 63650 Corps, Malan, Xinjiang Uygur Autonomous Region 841700, China.; 3 Department of Cardiovascular Medicine, First Affiliated Hospital of Medical School, Xi'an Jiaotong University, Xi'an, Shaanxi 710061, China.; 4 Department of Biomedical Informatics and Center for Quantitative Sciences, Vanderbilt University Medical Center, Nashville, Tennessee 37232, USA.; 5 International Cancer Center, Shenzhen University Health Science Center, Shenzhen, Guangdong 518060, China.; 6 Comprehensive Cancer Center, The Ohio State University, Columbus, Ohio 43210, USA.; 7 Department of Medicine, Vanderbilt University Medical Center, Nashville, Tennessee 37232, USA.; 8 State Key Laboratory of Translational Medicine and Innovative Drug Development, Jiangsu Simcere Diagnostics Co., Ltd., Nanjing, Jiangsu 210042, China.; 9 National Institute of Biological Sciences, Beijing 102206, China.

**Keywords:** miR-139-5p, KRAS mutation, CRC, Ras signaling, Wnt/β-catenin signaling

## Abstract

**Introduction:** Colorectal cancer (CRC) frequently harbors KRAS mutations that result in chemoresistance and metastasis. MicroRNAs (miRNAs) are usually dysregulated and play important regulatory roles in tumor progression. However, the KRAS mutation-responsive miRNA profile in CRC remains uninvestigated.

**Methods:** miR-139-5p was identified and evaluated by small RNA sequencing, qRT-PCR and *in situ* hybridization. The roles of miR-139-5p in CRC cells with and without KRAS mutation were determined by Cell Counting Kit-8 (CCK-8), colony formation, flow cytometry and transwell assays *in vitro* and by tumorigenesis and metastasis assays* in vivo*. Microarrays followed by bioinformatic analyses, luciferase reporter assays and Western blotting were applied for mechanistic studies.

**Results:** miR-139-5p was significantly downregulated in KRAS-mutated CRC cells and tissues compared with their wild-type counterparts. Low miR-139-5p expression was associated with aggressive phenotypes and poor prognosis in CRC patients. miR-139-5p overexpression inhibited CRC cell proliferation, migration and invasion* in vitro*, sensitized tumors to chemotherapy, and impaired tumor growth and metastasis *in vivo*. Transcriptomic profiling identified multiple modulators in the Ras (JUN and FOS) and Wnt (CTNNB1 and DVL1) signaling pathways and the epithelial-to-mesenchymal transition (EMT) process (ZEB1) as direct targets of miR-139-5p, and inverse correlations were confirmed in CRC clinical tissues. Aberrantly activated Wnt signaling in KRAS-mutant cells was demonstrated to transcriptionally repress miR-139-5p through TCF4, forming a miR-139-5p/Wnt signaling double-negative feedback loop.

**Conclusions:** We identified miR-139-5p as a KRAS-responsive miRNA and demonstrated its involvement in CRC progression. KRAS mutation disrupted the miR-139-5p/Wnt signaling reciprocal negative feedback mechanism, which might cause miR-139-5p downregulation and derepression of oncogenic signaling pathways and EMT. These results reveal a transcriptional regulatory mode of KRAS-driven malignant transformation and highlight miR-139-5p as a novel regulator of crosstalk between the Ras and Wnt signaling pathways in CRC.

## Introduction

Colorectal cancer (CRC) remains the second leading cause of cancer-related death worldwide 1. Although screening and removal of premalignant lesions have reduced the mortality of early CRC, the overall survival of advanced CRC with metastases and chemoresistance remains poor 2. Approximately 40% of CRC cases exhibit KRAS mutations, most of which occur in codons 12 and 13, arise early during CRC development and are maintained throughout CRC progression 3. KRAS mutations are GAP-insensitive, which renders the proteins constitutively GTP bound and activated, leading to persistent activation of oncogenic signaling pathways and downstream oncogenes and thus promoting cell proliferation and survival 4. Clinical investigations have revealed that CRC patients with KRAS mutations have more distant metastases, shorter survival times and poorer prognoses than patients with wild-type (WT) KRAS 5, 6. These observations might be partially explained by findings that KRAS mutation can drive CRC cell invasion and metastasis by inducing the expression of genes related to invasion, epithelial-to-mesenchymal transition (EMT) and stemness 7-9. Although KRAS mutations play important roles in CRC development and progression, the mechanisms underlying the KRAS-mediated promotion of CRC, especially with regard to transcriptional regulation, are still unclear.

MicroRNAs (miRNAs) are a class of 22-24-nucleotide noncoding RNAs that bind to complementary target mRNAs to degrade or inhibit their translation; this process is an important mode of posttranscriptional regulation 10. Accumulating evidence has demonstrated that miRNAs are frequently dysregulated and play key roles in cancer development and progression 10. Recent studies have indicated that genetic mutations in known transcriptional regulators contribute to miRNA dysregulation 11-13. For example, P53 mutation contributes to the chemoresistance of breast cancer cells by reducing the transcriptional activity of miR-223 14. APC mutation downregulates miR-155 expression, which reduces the survival and clonogenic capacity of CRC cells 15. Notably, although KRAS mutations are some of the most frequent genetic mutations in human cancers 3, particularly in CRC, the dysregulated miRNAs and their functions and underlying mechanisms following KRAS mutation have yet to be fully explored.

Dysregulation of miR-139-5p has been reported to occur in various malignancies, including breast cancer, liver cancer, parathyroid cancer, head and neck cancer, and CRC 16-21. Several lines of evidence have elucidated regulatory mechanisms of miR-139-5p expression. For example, P53 has been found to bind to the promoter of miR-139-5p and transactivate its expression, which suppresses PDE4D and cAMP signaling and inhibits cancer cell growth 21. miR-139-5p is downregulated by adsorption to the long noncoding RNA LINC00152 in gastric cancer (GC), which facilitates cancer cell glycolysis by regulating PRKAA1 22. We previously found that CD44 induces deacetylation of histone H3 lysine 9 to suppress miR-139-5p transcription, thereby promoting cell growth and invasion in GC, indicating that epigenetic modifications are involved in miR-139-5p dysregulation 23. Using small RNA sequencing in a pair of KRAS isogenic CRC cell lines, we have found that miR-139-5p is significantly downregulated in KRAS-mutant CRC cells. However, the cause of miR-139-5p dysregulation associated with KRAS mutation in CRC remains undetermined.

In this study, we verified that miR-139-5p is frequently downregulated in KRAS-mutant CRC cells and tissues compared with their WT counterparts and found that miR-139-5p overexpression inhibits CRC cell proliferation, migration and invasion* in vitro* and tumorigenesis and metastasis *in vivo*. miR-139-5p directly targets multiple modulators in the Ras and Wnt signaling pathways and the EMT process. Furthermore, we found a miR-139-5p/Wnt signaling reciprocal negative feedback loop in KRAS-mutant cells. Identification of these new mechanisms underlying KRAS mutation-driven malignancy may promote the development of prognostic biomarkers and potential therapeutics for CRC.

## Materials and Methods

### Cell culture and reagents

Caco-2, SW48, COLO320DM, NCI-H716, HuTu80, SW480, HCT8, SW837, HCT116, SK-CO-1, LoVo, DLD-1, SW1116, SW1463, SW948, SW620, LS174T, T84 and LS123 cells were obtained from the American Type Culture Collection (ATCC). DKs-8 and DKO-1 isogenic cells were obtained from the Robert Coffey Lab (Vanderbilt University). The V9P cell line was provided by the Mariadason Lab (Olivia Newton-John Cancer Research Institute). The cells were maintained in a humidified atmosphere with 5% CO_2_ at 37°C. All cell lines were authenticated by short tandem repeat analysis using an AmpF/STR Identifier Kit (Applied Biosystems). Recombinant human Wnt3a (R&D Systems) was dissolved in PBS containing 0.1% BSA. ICG-001 (Santa Cruz Biotechnology, Santa Cruz) was dissolved in DMSO.

### Small RNA sequencing analysis

Total RNA was extracted from each sample, and approximately 1 µg of each RNA sample was used to establish a small RNA library using an NEBNext Small RNA Library Prep Set for Illumina (New England Biolabs). The PCR products were purified using a QIAquick PCR Purification Kit (Qiagen). The products were accurately quantified for sequencing applications using a quantitative real-time PCR (qRT-PCR)-based KAPA Biosystems Library Quantification Kit. Single-end sequencing (50 bp) was performed on a NextSeq 500 Sequencer (Illumina). Mapped reads were annotated, and miRNA expression was quantified using ncPRO-seq (v1.5.1) 61 based on miRbase v19. Differentially expressed miRNAs between DKO-1 and DKs-8 cells were examined using edgeR 60. Differential expression was determined based on fold change (FC) and P values, with |log 2 (FC)|>1 and P<0.01.

### qRT-PCR

An RNeasy Plus Mini Kit (50 reactions) (Qiagen, Hilden, Germany) was used to extract total RNA, which was then reverse transcribed with an Advantage RT-for-PCR Kit (Qiagen). The target sequence was amplified via qRT-PCR with a SYBR Green PCR Kit (Qiagen). The 2^-ΔΔCt^ method was used to determine the relative fold changes (FCs) in target gene expression in the cell lines normalized to the levels in corresponding control cells (defined as 1.0). U6 small nuclear RNA and GAPDH were used as internal controls for miRNA and mRNA assays, respectively. In the 2^-ΔΔCt^ equation, ΔCt=ΔCt target-ΔCt U6/GAPDH, and ΔΔCt=ΔCt expression vector-ΔCt control vector. All experiments were performed in duplicate. The PCR primers for miR-139-5p and U6 were purchased from RiBoBio (Guangzhou, China); the other PCR primers are listed in Supplementary Table 3.

### Human tissue collection

A commercial tissue microarray containing samples from 20 primary CRC tissues and 80 pairs of CRC and adjacent normal tissues was purchased from Outdo Biotech Company (Shanghai, China). Sixty paired primary CRC tissues and adjacent nontumor tissues were collected from CRC patients who had undergone surgery at Xijing Hospital. Informed consent was obtained from each patient. All procedures in this study were approved by the Protection of Human Subjects Committee of Xijing Hospital.

### Targeted Sanger sequencing of KRAS

Mutant KRAS alleles were sequenced by targeted Sanger sequencing. We isolated genomic DNA using a QIAamp DNA FFPE Tissue Kit (Qiagen), and the PCR products were then sequenced using a BigDye Terminator 3.1 Cycle Sequencing Kit (Applied Biosystems) following the manufacturer's protocols.

### *In situ* hybridization (ISH) and immunohistochemistry (IHC)

Sections of samples that were 4 μm thick were embedded in paraffin and used for ISH and IHC. For IHC, slides were probed with primary antibodies against JUN, FOS, β-catenin, DVL1, and ZEB1 that were purchased from Cell Signaling Technology (CST). An antibody against Ki-67 was purchased from Santa Cruz. For ISH, slides were incubated with 5'- and 3'-digoxigenin-labeled locked nucleic acid-based miR-139-5p probes (Exiqon). Then, the slides were incubated with horseradish peroxidase-conjugated secondary antibodies (Dako) and stained with DAB chromogenic substrate (Dako). IHC and ISH staining was scored by two independent observers as previously described. Ki-67 staining was quantified by counting the positively stained nuclei per field. miR-139-5p, JUN, FOS, β-catenin, DVL1, and ZEB1 staining was quantified based on the intensity (0, no staining; 1, weak staining; 2, moderate staining; and 3, strong staining) and extent (0, no positive tumor cells; 1, <10%; 2, 10-50%; and 3, >50%) of staining. The staining index (SI) for each specimen was calculated as the product of the staining intensity and the percentage of positive tumor cells. Samples with an SI≥4 were determined to have high expression, and samples with an SI<4 were determined to have low expression.

### Plasmid construction

Expression plasmids for β-catenin, TCF3 and TCF4 were constructed by inserting the corresponding cDNA sequences into the pcDNA3.1 vector; 3'-UTR luciferase reporter plasmids for JUN, FOS, DVL1, CTNNB1, TCF4 and ZEB1 were constructed by inserting the WT 3'-UTR sequences of the corresponding genes into the psiCHECK-2 Luciferase vector. Mutant constructs were created by mutating the seed regions of the miR-139-5p-binding site. Promoter luciferase reporter constructs were constructed by inserting the -5000 to +1 sequence of the 5'-flanking region of the *MIR139* from human genomic DNA into the pGL3-Basic vector. A QuikChange II Site-Directed Mutagenesis Kit (Stratagene) was used to mutate the TCF4 binding sites.

### Transient transfection and lentiviral infection

A synthetic miR-139-5p agomir, antagomir and corresponding negative controls were purchased from RiBoBio. siRNAs against TCF3, TCF4, and β-catenin and their scrambled controls were purchased from GenePharma (Shanghai, China). Lentiviruses expressing miR-139-5p or short hairpin RNAs against miR-139-5p sequences were obtained from GeneChem (Shanghai, China). To generate stable cell lines, the indicated cells were infected with lentiviruses at a multiplicity of infection of 100:1. Infection efficiency was confirmed by qRT-PCR at 72 h after infection, and the cells were selected with puromycin for 2 weeks.

### Cell Counting Kit-8 (CCK-8) assay

Cells were seeded into 96-well plates at a density of 1000 cells in 100 μL of complete medium per well. At each time point, the original medium was replaced with a 1:9 mixture of CCK-8 solution (Transgene) and complete medium, and the cells were then incubated at 37°C for 2 h. The absorbance of each sample at 450 nm was analyzed by a microplate reader (Tecan), and each sample was measured three times.

### Colony formation assay

Cells were seeded into 6-well plates at a density of 1000 cells in 2 mL of complete medium per well. After 14-18 d of culture, the cells formed stable colonies. The cell colonies were fixed with 70% ethanol and then stained with a crystal violet solution. Colonies containing more than 50 cells were counted, and each group had three replicates.

### Cell cycle and apoptosis assays

Transfected cells were fixed in 75% ethanol and stained with propidium iodide (Sigma-Aldrich) supplemented with RNase A for cell cycle analysis. An Annexin V-FITC Apoptosis Detection Kit (BD Biosciences) was used for apoptosis assays according to the manufacturer's protocol. Cells were sorted using a fluorescence-activated cell sorter (BD).

### Transwell migration and invasion assays

For invasion assays, chamber inserts with an 8-µm pore size were first coated with 200 mg/mL Matrigel (Corning), and the uppermost chamber was plated with 1×10^5^ cells. For cell migration assays, the upper chamber with a noncoated membrane was plated with 5×10^4^ cells. Each assay was repeated three times, and three different inserts were used to obtain the mean number of cells in five fields per membrane.

### *In vivo* tumor growth and metastasis assays

All animal procedures were approved by the Fourth Military Medical University Animal Care Committee. BALB/c nude mice (6-8 weeks old) were used based on the institutional guidelines for animal care. For tumor growth models, luciferase-tagged cells were subcutaneously injected into the flanks of mice (6×10^6^ cells/100 µL of PBS per injection site; 10 mice per group). The tumor volume was calculated with the following formula: tumor maximum diameter (L)×diameter along the perpendicular axis (W)^2^/2. When the tumor sizes reached approximately 100 mm^3^, the mice were randomized into treatment and control groups. Fluorouracil (5-FU) was intraperitoneally injected at a dosage of 8 mg/kg/d. For tumor metastasis models, luciferase-tagged cells (4×10^6^ cells/100 µL of PBS) were injected into the tail veins or spleens of nude mice (10 mice per group). The survival of all mice was recorded throughout the experiment. Firefly luciferase was used for *in vivo* tracking of tumor formation and metastasis. The bioluminescence signals in tumor-bearing mice were detected using an *in vivo* imaging system (PerkinElmer).

### Immunoblotting analysis

Protein lysates were separated by SDS-PAGE and transferred onto nitrocellulose membranes. The membranes were incubated for 1 h with a mixture of a Tween 20 solution (0.05% Tween 20, 150 mM NaCl and 120 mM Tris-HCl [pH 7.4]) and 5% milk in Tris-buffered saline (Tris-buffered saline with Tween 20, TBST) at room temperature to inhibit nonspecific binding reactions. After incubation with primary and secondary HRP-conjugated antibodies in 5% nonfat milk, the immunoreactive proteins were detected with Dura SuperSignal Substrate (Pierce). Antibodies against β-actin, JUN, FOS, β-catenin, DVL1, ZEB1, E-cadherin, Fibronectin and Vimentin were purchased from CST.

### Immunofluorescence

Cells were plated onto glass coverslips, fixed with 4% paraformaldehyde and permeabilized with 0.1% Triton X-100 in PBS. The cells were incubated with primary antibodies against Vimentin (CST) and E-cadherin (CST) at 4°C overnight and were then incubated with FITC-conjugated goat anti-rabbit and Cy3-conjugated goat anti-mouse secondary antibodies for 2 h at room temperature. The immunostaining signals and DAPI-stained nuclei were visualized at room temperature using a confocal microscope (FV10i; Olympus).

### Terminal deoxynucleotidyl transferase dUTP nick-end labeling (TUNEL) assay

A TUNEL assay was performed to assess apoptosis using an *In situ* Cell Death Detection Kit, POD (Roche), following the manufacturer's protocols. Slides were fixed in 4% paraformaldehyde for 1 h at room temperature, processed for TUNEL staining, washed with PBS, labeled with Hoechst 33258 (10 µg/µL), rinsed with PBS and mounted with a propyl gallate solution. A labeling solution without the enzyme was added as the negative control.

### Agilent cDNA microarray analysis

DKO-1 cells were transfected with 20 nM miR-139-5p or control agomir. Twenty-four hours after transfection, the cells were harvested to detect changes at the mRNA level. Four hundred nanograms of total RNA was amplified and labeled using a Low Input Quick Amp Labeling Kit (Agilent Technologies) and hybridized onto Agilent Whole Human Genome Oligonucleotide Microarrays. The expression data were preprocessed and normalized by the quantile algorithm in Gene Spring Software v11.0.

### Luciferase reporter assay

Luciferase assays were performed using a Dual-Luciferase Reporter Assay System (Promega) according to the manufacturer's protocol. Firefly luciferase activity was normalized to Renilla luciferase as an internal control. The transfection experiments were performed in triplicate for each plasmid construct.

### Chromatin immunoprecipitation (ChIP)

ChIP assays were performed using a Magna ChIP G Assay Kit (EMD Millipore). Cells were crosslinked with 1% formaldehyde for 10 min at room temperature and quenched in glycine. DNA was immunoprecipitated from the sonicated cell lysates using a TCF4 antibody (CST) and subjected to PCR to amplify the TCF4 binding sites. The amplified fragments were then analyzed by agarose gel electrophoresis. A nonspecific antibody against IgG (BD) served as the negative control. The primers used in the ChIP assays are listed in Supplementary Table 3.

### Bioinformatics analysis

The Kyoto Encyclopedia of Genes and Genomes (KEGG) database was used to analyze the main biological functions of the differentially expressed genes. The CluGO plugin of Cytoscape was used to determine the enriched Gene Ontology (GO) terms and signaling pathways of the differentially expressed genes. The Benjamini-Hochberg algorithm was used for *P* value correction. Enrichment was considered significant at a Q value ≤ (corrected P value) ≤0.05. Gene set enrichment analysis (GSEA) was performed using GSEA software v2.07. The enrichment score (ES) was calculated to assess the overrepresentation of members of a predefined gene set appearing at the extremes (top and bottom) of the ranked gene list. Oncomine and The Cancer Genome Atlas (TCGA) datasets were used to determine the expression of miR-139-5p in human cancer specimens compared with normal tissues. TCGA data were analyzed using R studio (v3.5.1).

### Statistical analysis

All statistical analyses were carried out with SPSS software (v19.0). Fisher's exact test was used for categorical data, while Student's *t*-test was used for intergroup comparisons for quantitative data. Kaplan-Meier survival analysis was used to determine cumulative survival rates. The statistical significance threshold was defined as *P*<0.05.

## Results

### miR-139-5p is downregulated in KRAS-mutant CRC cells and tissues

**Figure 1:**
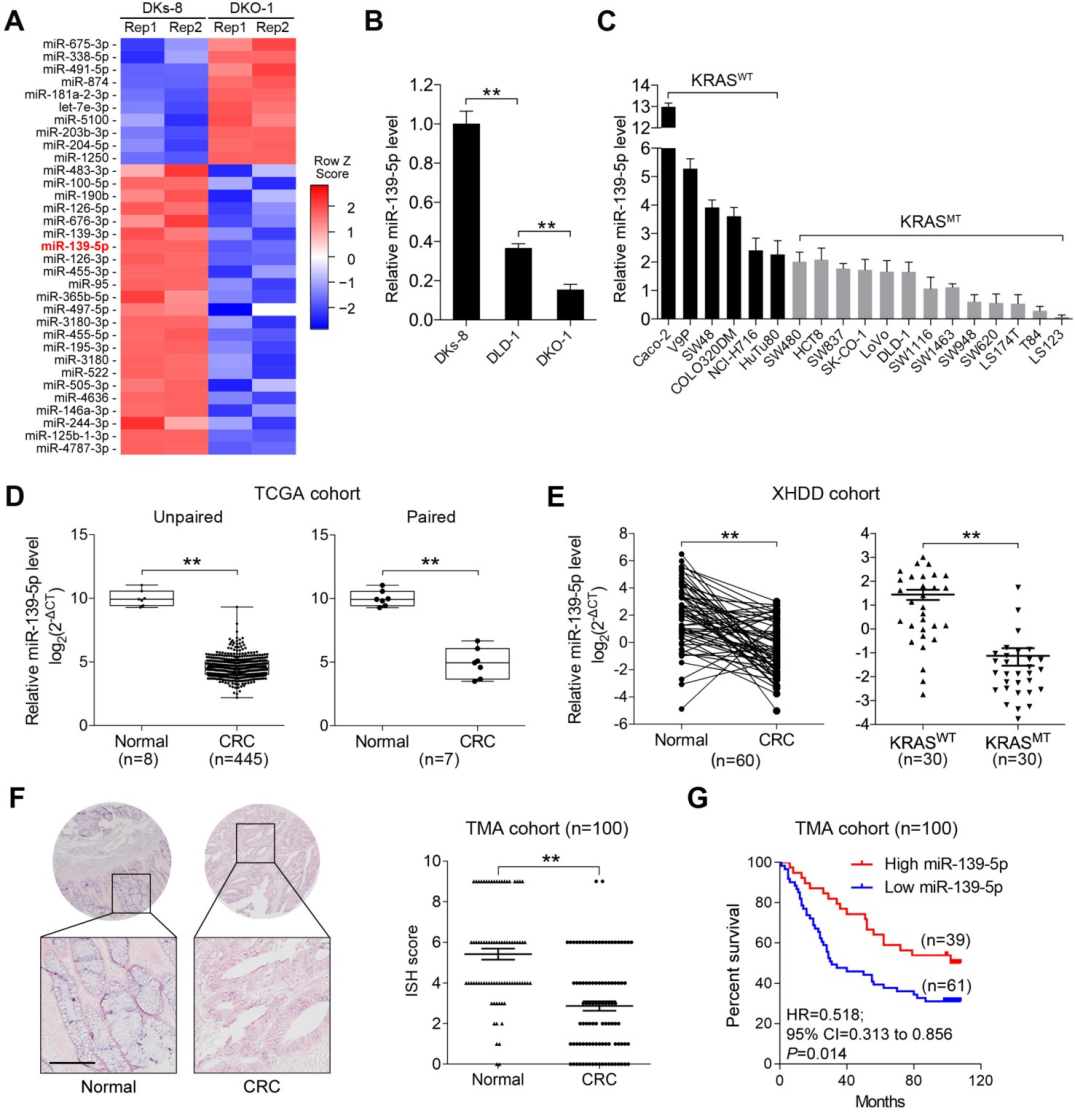
** miR-139-5p is downregulated in KRAS-mutant CRC cells and tissues. (A)** miRNA heatmap showing the altered miRNAs (|log2(FC)|>1 and P<0.01) in DKO-1 (mutant KRAS) versus DKs-8 (WT KRAS) cells. **(B)** The expression level of miR-139 were examined in DLD-1 isogenic cell lines using qRT-PCR. **(C)** Expression level of miR-139 in a panel of CRC cell lines grouped based on their expression of WT KRAS (KRAS^WT^) versus mutant KRAS (KRAS^MT^). **(D)** Box plots showing the expression of miR-139-5p in CRC tissues compared with unpaired (left) or paired (right) adjacent normal tissues in TCGA cohorts. **(E)** Left, qRT-PCR results showing miR-139-5p level in 60 pairs of matched human CRC specimens and adjacent normal tissues in the XHDD cohort. Right, miR-139-5p level in WT and KRAS-mutant CRC tissues from the XHDD cohort. Each symbol represents the mean value of an individual patient. U6 snRNA served as the internal control. Independent experiments were performed in triplicate. **, *P*<0.01.** (F)** Representative ISH images are shown. The ISH scores showed that miR-139-5p expression was downregulated in CRC tissues compared with adjacent normal tissues. **(G)** Kaplan-Meier overall survival curves for CRC patients with low or high expression of miR-139-5p.

To identify miRNAs affected by KRAS mutation in CRC, we utilized isogenic derivatives of DLD-1 cells that contain one WT (DKs-8 cells) or one G13D mutant KRAS allele (DKO-1 cells) 24. The DKO-1 cells were genetically engineered to express only the mutant KRAS allele, and the DKs-8 cells expressed only WT KRAS. Via small RNA sequencing, we found that 10 miRNAs were upregulated and 23 miRNAs were downregulated in DKO-1 cells compared to DKs-8 cells (|log2(FC)|>1, *P*<0.01, Figure 1A). miR-139-5p was selected from the downregulated miRNAs in KRAS-mutant cells for further investigation because it has been reported to be a tumor suppressor in several cancer types 16, 17, 25, yet its relation to KRAS mutation remains unclear. qRT-PCR confirmed that miR-139-5p expression was reduced in DKO-1 cells compared with DKs-8 cells (Figure 1B). Furthermore, in a panel of CRC cell lines with WT or mutant KRAS, we found lower miR-139-5p levels in KRAS-mutant cells than in WT cells (Figure 1C). To evaluate the clinical significance of miR-139-5p, we analyzed TCGA data and found that miR-139-5p was significantly downregulated in CRC tissues relative to normal tissues; a similar trend was observed in paired CRC relative to adjacent normal samples (Figure 1D). The downregulation of miR-139-5p was further validated in an independent cohort of 60 pairs of CRC and adjacent normal samples from Xijing Hospital of Digestive Diseases (XHDD, Figure 1E and Supplementary Table 1). Notably, among CRC tissues, lower miR-139-5p levels were observed in KRAS-mutant tissues than in WT tissues (Figure 1E). Moreover, ISH of a tissue microarray (TMA) cohort including 20 cases of primary CRC tissues and 80 pairs of CRC and adjacent normal tissues in KRAS-mutant CRC tissues (Figure 1F). Further correlation analyses showed that lower miR-139-5p expression was associated with a more aggressive phenotype in CRC (Table 1). Kaplan-Meier analysis revealed poor survival of CRC patients with low miR-139-5p expression (Figure 1G). Univariate and multivariate analyses indicated that low miR-139-5p expression may serve as an independent risk factor for CRC prognosis (Table 2). These findings suggest that miR-139-5p might be a clinically relevant KRAS-regulated miRNA in CRC.

### miR-139-5p inhibits proliferation, migration and invasion in KRAS-mutant CRC cells

**Figure 2:**
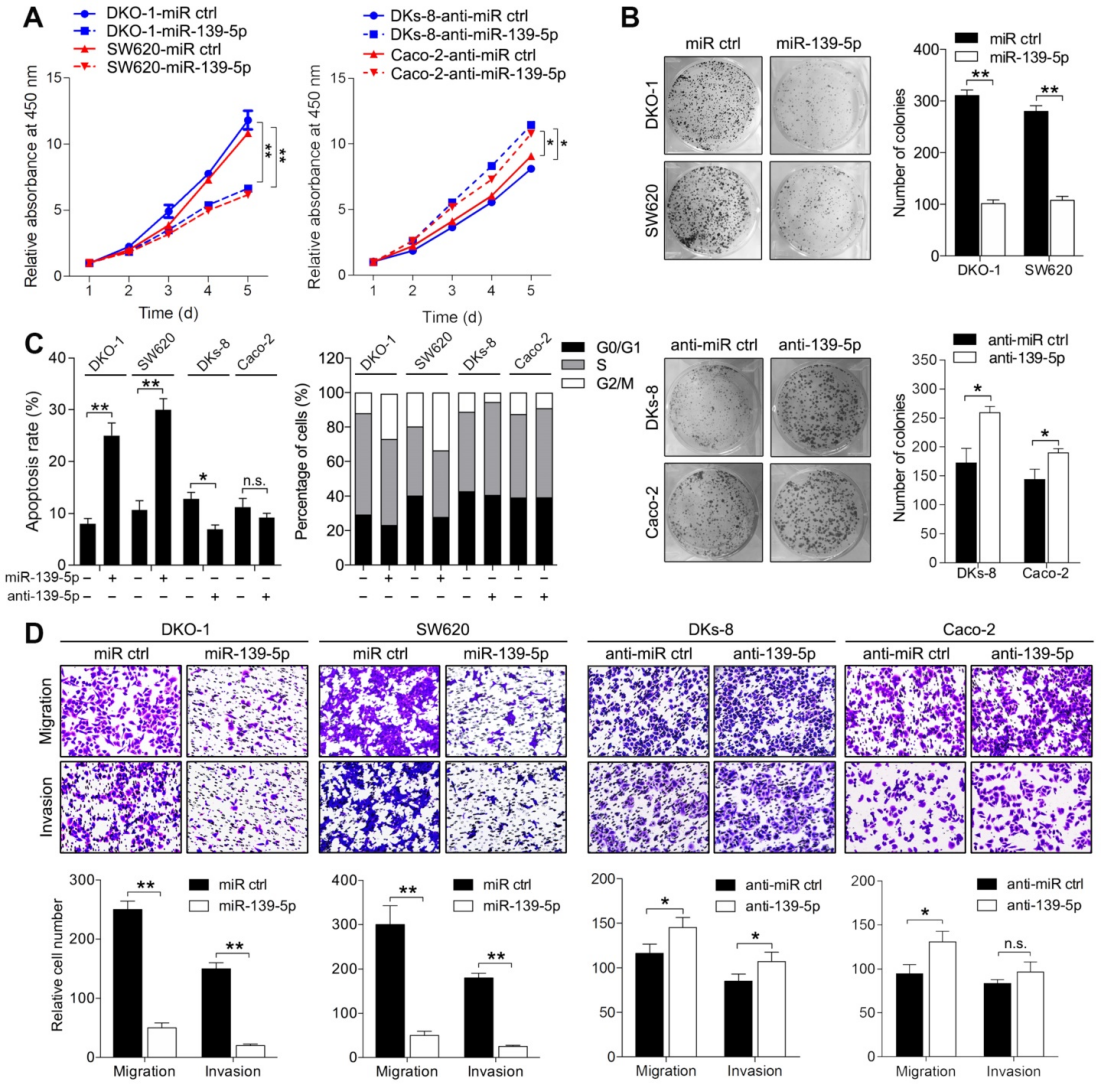
** miR-139-5p inhibits CRC proliferation, migration and invasion *in vitro*. (A)** Growth curves of CRC cells transfected with an agomir (miR-139-5p) or antagomir (anti-miR-139-5p) of miR-139-5p or their negative controls, as revealed by CCK-8 analyses. **(B)** Colony formation assays were performed on CRC cells transfected with miR-139-5p, anti-miR-139-5p, or corresponding negative controls. **(C)** Apoptosis rates and cell cycle distributions of CRC cells transfected with miR-139-5p, anti-miR-139-5p, or their negative controls. **(D)** Transwell migration and invasion assays were performed on CRC cells transfected with miR-139-5p, anti-miR-139-5p, or their negative controls. The results are representative results (n=3) from experiments performed in triplicate. The results are expressed as the means±SDs. **, *P*<0.01.

To determine the phenotypes resulting from miR-139-5p loss in mutant RAS cells, we transfected KRAS-mutant DKO-1 and SW620 cells with a miR-139-5p agomir and introduced a miR-139-5p antagomir into WT KRAS-expressing DKs-8 and Caco-2 cells. The inhibition or overexpression of miR-139-5p in the corresponding cells upon transfection was confirmed by qRT-PCR (Supplementary Figure S1A). CCK-8 assays and colony formation assays were performed to examine the effect of miR-139-5p on cell proliferation capacity. Overexpression of miR-139-5p in DKO-1 and SW620 cells significantly reduced proliferation and colony-forming efficiency. In contrast, inhibition of miR-139-5p in DKs-8 and Caco-2 cells increased cell growth and colony formation (Figure 2A and 2B). Flow cytometry analyses showed that miR-139-5p overexpression substantially increased apoptosis and induced G2/M phase cell cycle arrest, while miR-139-5p inhibition with the antagomir suppressed apoptosis and facilitated cell cycle progression (Figure 2C). Transwell assays showed that miR-139-5p overexpression markedly suppressed migration and invasion in DKO-1 and SW620 cells, while miR-139-5p inhibition increased the migratory and invasive abilities of DKs-8 and Caco-2 cells (Figure 2D). These results indicate that miR-139-5p plays tumor-suppressive roles in CRC cells, opposing the effects of KRAS mutation.

### miR-139-5p increases responsiveness to chemotherapy and suppresses metastasis in KRAS-mutant CRC cells

**Figure 3:**
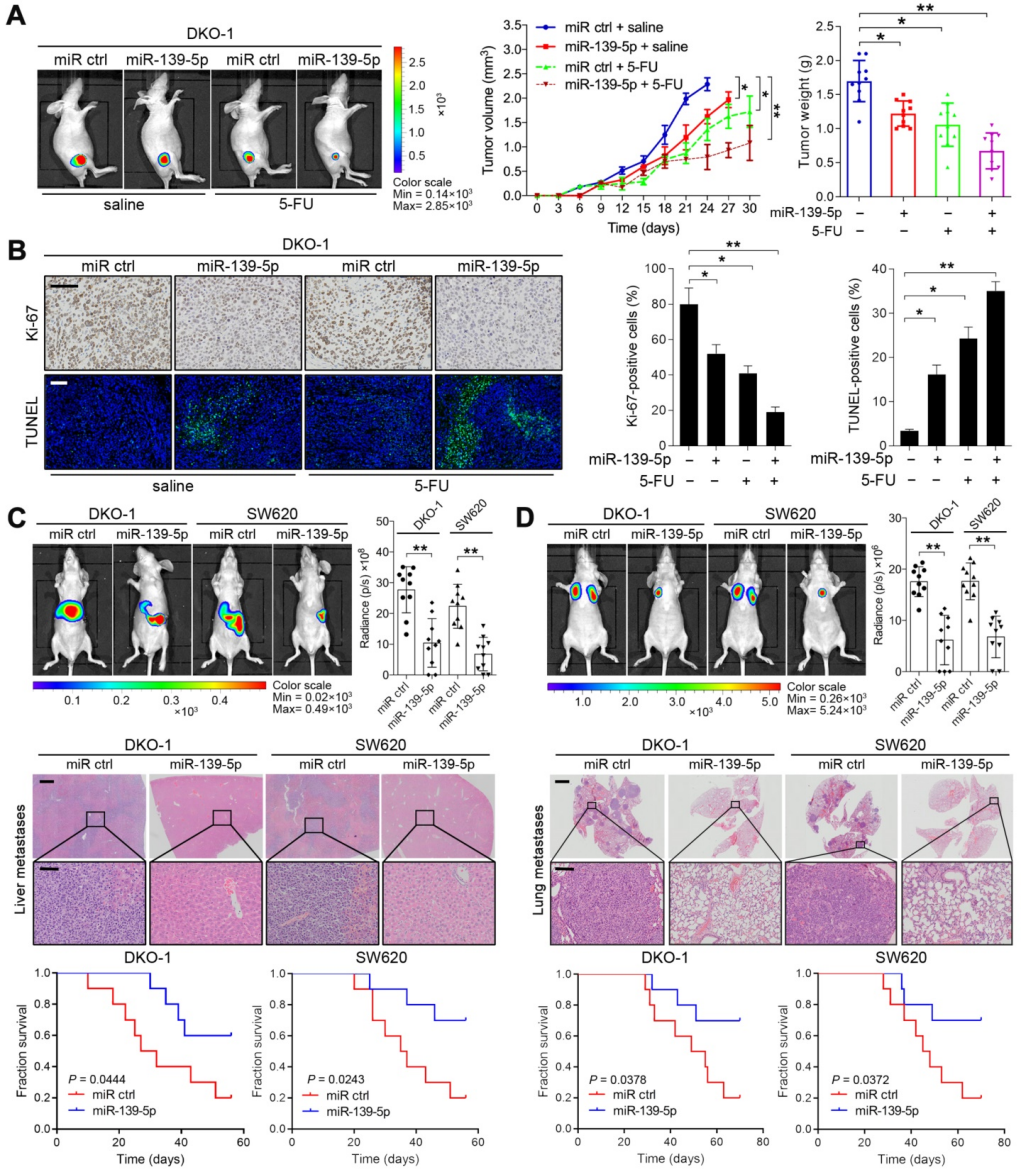
** miR-139-5p suppresses CRC tumorigenesis and metastasis *in vivo*. (A)** The indicated cells were injected subcutaneously into nude mice (n=10). After the tumor size reached approximately 100 mm^3^, the mice received 5-FU treatment (8 mg/kg/d, i.p. injection). Left, representative bioluminescent images captured from subcutaneous tumors. Middle, growth curves of tumors in nude mice injected with the indicated cells. Right, calculated weights and volumes of tumors isolated on day 30 after treatment. **P<0.01 by one-way ANOVA followed by Dunnett's test compared with the miR ctrl group. **(B)** Left, representative images of tumor samples that were stained via IHC for Ki-67 and TUNEL staining. Right, the percentages of Ki-67- and TUNEL-positive cells. Bars: 200 µm. **(C)*** In vivo* liver metastasis assay results. Top, representative bioluminescent images and radiance levels of liver metastases in the different groups at 8 weeks after intrasplenic inoculation. Middle, representative hematoxylin and eosin (H&E) staining showing metastatic nodules in liver tissues of nude mice. Bars: 200 µm (upper); 50 µm (lower). Bottom, overall survival times of the nude mice in the different groups. **(D)*** In vivo* lung metastasis assay results. Top, representative bioluminescent images and radiance levels of lung metastases in the different groups at 8 weeks after tail vein injection. Middle, representative H&E staining showing metastatic nodules in the lungs of nude mice. Bars: 1000 µm (upper); 50 µm (lower). Bottom, overall survival times of the nude mice in the different groups. The data are presented as the means±SDs. **, *P*<0.01; n.s., not significant.

To extend the above findings to an *in vivo* setting, stable miR-139-5p-overexpressing DKO-1 and SW620 cells were established by lentivirus infection and then subcutaneously injected into the flanks of nude mice. Increased expression of miR-139-5p upon transfection was confirmed by real-time PCR (Supplementary Figure S1B). Tumor size and tumor weight were significantly lower in mice implanted with miR-139-5p-overexpressing cells than in control mice (Figure 3A and Supplementary Figure S1C-D). 5-FU, a standard chemotherapeutic agent for CRC treatment, was injected intraperitoneally after the tumor volume reached approximately 100 mm^3^. Treatment with 5-FU impaired tumor growth, and the inhibitory effect was more pronounced when miR-139-5p was overexpressed (Figure 3A and Supplementary Figure S1C-D). Furthermore, xenografts derived from miR-139-5p-overexpressing cells exhibited decreased proliferation and increased apoptosis, as evidenced by Ki-67 and TUNEL staining (Figure 3B and Supplementary Figure S1E).

The liver and lung are the most common metastatic sites for CRC 26. To determine the role of miR-139-5p in metastasis, we established liver and lung metastasis models by injecting miR-139-5p-overexpressing DKO-1 and SW620 cells into the spleens and tail veins of nude mice, respectively. Compared to the control condition, overexpression of miR-139-5p decreased the incidence of liver metastasis and the number of metastatic nodules and improved survival in mice (Figure 3C). Similarly, miR-139-5p overexpression reduced lung metastasis following tail vein injection (Figure 3D). These results indicate that miR-139-5p retards tumor progression and reinforces responsiveness to chemotherapy in KRAS-mutant CRC cells.

### High-throughput screening and identification of miR-139-5p targets in CRC cells

**Figure 4:**
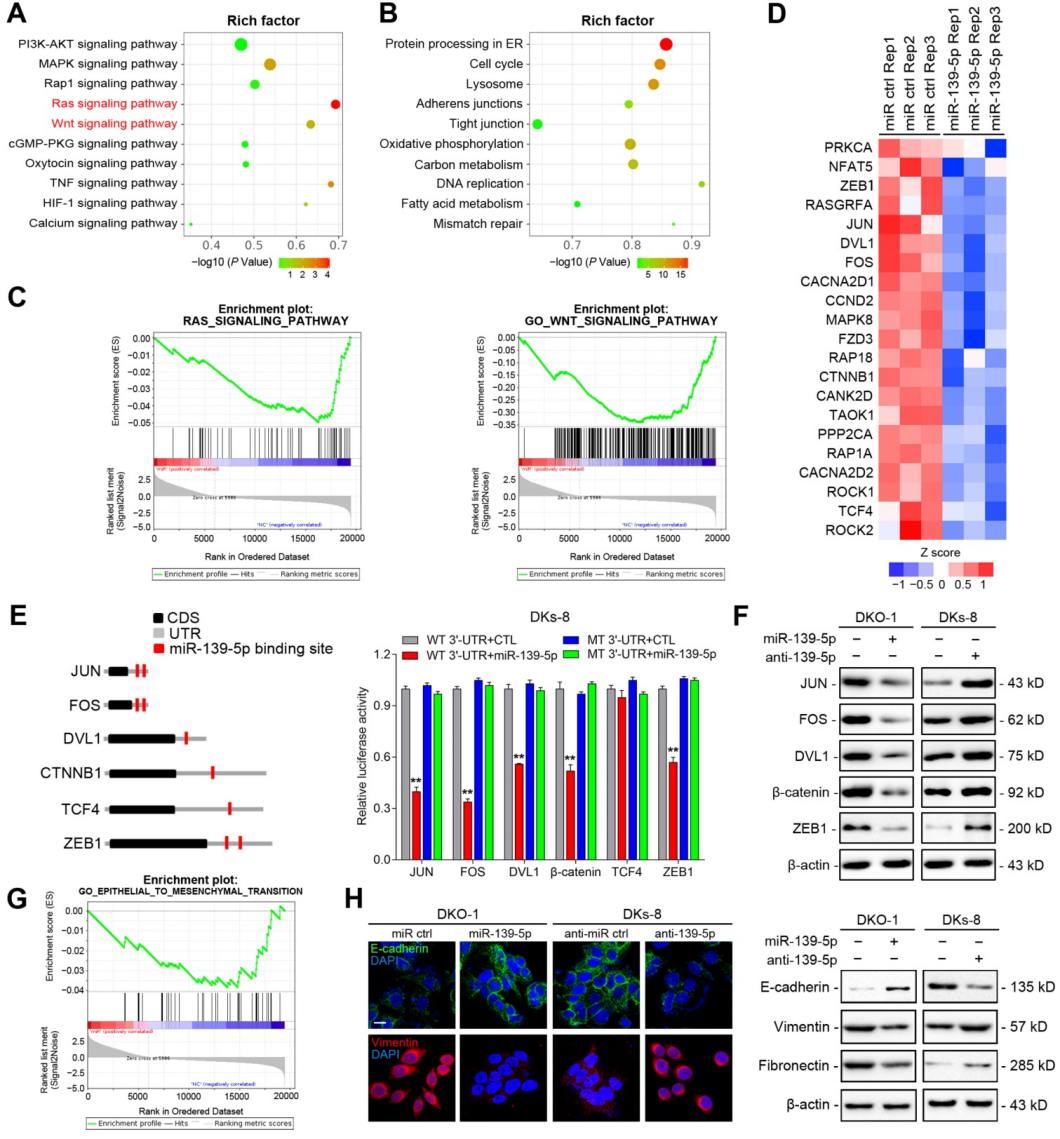
** High-throughput screening and identification of miR-139-5p targets in CRC cells. (A)** Major signaling pathways and **(B)** cellular processes suppressed by miR-139-5p expression in DKO-1 cells, as revealed by KEGG pathway analysis. **(C)** GSEA revealed negative enrichment of miR-139-5p-altered genes in the RAS (left) and Wnt (right) signaling gene sets. **(D)** Heatmap of representative RAS- or Wnt-related genes that were predicted to possess miR-139-5p binding sites from the DKO-1 cell transcriptomic results. **(E)** Left, predicted miR-139-5p binding sites in the 3'-UTRs of human JUN, FOS, DVL1, CTNNB1, TCF4 and ZEB1. CDS, coding sequence. Right, luciferase activity derived from the indicated 3'-UTR reporter constructs after cotransfection of DKO-1 cells with miR-139-5p or negative controls. The results show the means±SDs (error bars) for three experiments performed in triplicate. **(F)** Immunoblots of JUN, FOS, ZEB1, CTNNB1 and DVL1 levels in the indicated cells after miR-139-5p overexpression or inhibition.** (G)** EMT gene sets negatively enriched in miR-139-5p-overexpressing DKO-1 cells, as revealed by GSEA. **(H)** Left, immunofluorescence staining of E-cadherin and Vimentin in the indicated cells after miR-139-5p overexpression or inhibition. Right, immunoblots of E-cadherin, Vimentin and Fibronectin in the indicated CRC cells.

To identify functional targets of miR-139-5p in CRC, we performed gene expression microarray profiling in DKO-1 cells after miR-139-5p overexpression and identified 3878 differentially expressed genes (|log2(FC)|>1, *P*<0.01). KEGG analysis revealed that the Ras and Wnt signaling pathways were the most significantly affected pathways (Figure 4A). Biological process analysis suggested that miR-139-5p inhibited the cell cycle, focal adhesion and cellular adhesion (Figure 4B), consistent with our experimental results. GSEA revealed that the miR-139-5p-induced gene expression alterations were negatively associated with the KEGG Reactome terms 'RAS pathway' and 'Wnt signaling pathway' (Figure 4C). Among the differentially expressed genes, 370 were associated with RAS or Wnt signaling, and 21 of them contained miR-139-5p binding sites in their 3'-UTRs (Figure 4D and Supplementary Table 2). Among the 21 candidates, FOS and JUN are transcription factors (TFs) regulated by RAS-activated signaling 27; DVL1, TCF4 and CTNNB1 are key regulators in Wnt signaling 28; and ZEB1 is an important EMT regulator involved in Ras and Wnt signaling 29. We therefore selected these 6 genes for further validation. Using dual luciferase reporter assays, we confirmed that FOS, JUN, DVL1, CTNNB1 and ZEB1, but not TCF4, were direct targets of miR-139-5p in DKs-8 cells (Figure 4E). The immunoblotting results confirmed the regulation of the targets by miR-139-5p in DKO-1 and DKs-8 cells (Figure 4F).

EMT plays important roles in regulating metastasis and drug resistance 30. Given that miR-139-5p was found to directly target ZEB1, we examined whether miR-139-5p regulates EMT in CRC cells. GSEA indicated that miR-139-5p-induced gene expression changes were negatively correlated with EMT core gene expression (Figure 4G). Immunofluorescence and immunoblotting further showed that overexpression of miR-139-5p in DKO-1 cells significantly upregulated the epithelial marker E-cadherin and downregulated the mesenchymal markers Vimentin and Fibronectin (Figure 4H). In contrast, E-cadherin was downregulated, while Vimentin and Fibronectin were upregulated, following inhibition of miR-139-5p in DKs-8 cells (Figure 4H). These results indicate that miR-139-5p plays a tumor-suppressive role by targeting multiple genes in oncogenic pathways and regulating EMT in CRC cells.

### Wnt/β-catenin signaling transcriptionally represses miR-139-5p in KRAS-mutant CRC cells

**Figure 5:**
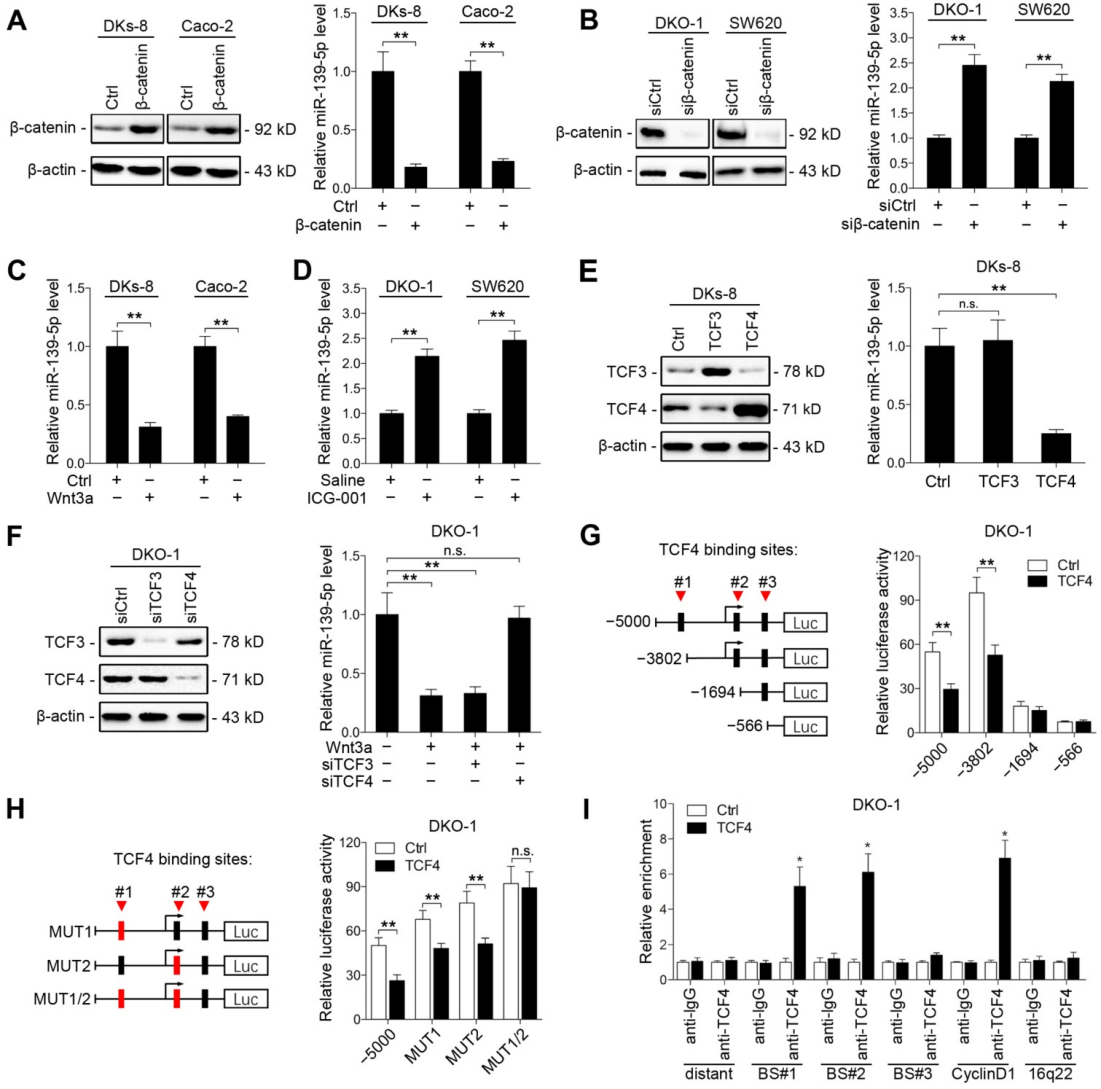
** Wnt/β-catenin signaling transcriptionally represses miR-139-5p in KRAS-mutant CRC cells. (A)** Left, immunoblots of β-catenin expression in DKs-8 and Caco-2 cells transfected with β-catenin and control plasmids. Right, miR-139-5p expression detected in the indicated cells by qRT-PCR. **(B)** Left, immunoblots of β-catenin expression in DKO-1 and SW620 cells transfected with β-catenin siRNA (siβ-catenin) or control siRNA (siCtrl). Right, miR-139-5p expression detected in the indicated cells by qRT-PCR. **(C)** DKs-8 and Caco-2 cells were treated with Wnt3a (5 ng/mL) or DMSO for 24 h. miR-139-5p expression was detected by qRT-PCR.** (D)** DKO-1 and SW620 cells were treated with ICG-001 (2 µg/mL) or DMSO for 24 h. miR-139-5p expression was detected by qRT-PCR.** (E)** DKs-8 cells were transfected with TCF3, TCF4, or control plasmid for 48 h. Immunoblotting was performed to examine the expression of TCF3 and TCF4. miR-139-5p expression was detected by qRT-PCR. **(F)** DKO-1 cells were transfected with TCF3 siRNA (siTCF3) and TCF4 siRNA (siTCF4) for 48 h. Immunoblotting was performed to examine the expression of TCF3 and TCF4. miR-139-5p expression was detected by qRT-PCR. **(G)** Left, schematic representation of consecutive deletion constructs spanning the promoter region (-5000 to +1) of *MIR139*, with the site -2373 nucleotides upstream of *MIR139* sequence as the transcription start site 23. The putative TCF4-binding sites in the miR-139 promoter region are shown in black boxes. Right, luciferase activity in DKO-1 cells transfected with the luciferase vector pGL3 driven by either the WT or deletion promoter.** (H)** Left, schematic representation of the mutation constructs of the miR-139 promoter. Right, luciferase activity in DKO-1 cells transfected with the luciferase vector pGL3 driven by either the WT or mutant promoter. **(I)** The ChIP assay demonstrated the direct binding of TCF4 to the miR-139 promoter in DKO-1 cells. qRT-PCR was carried out to quantitate the immunoprecipitated products using primers within the miR-139 promoter. Primers within the cyclin D1 and 16q22 promoters served as the positive and negative controls, respectively. *P< 0.05; **P <0.01; n.s., not significant.

Aberrant activation of Wnt signaling contributes to CRC development 31. Previous studies have reported that the KRAS G12V mutation promotes Wnt/β-catenin signaling activity 32, and miR-139-5p has been found to be upregulated in dominant-negative TCF4 cells 33. We thus investigated whether KRAS mutation-activated Wnt/β-catenin signaling represses miR-139-5p expression in CRC cells. We found that ectopic expression of β-catenin decreased miR-139-5p expression in DKs-8 and Caco-2 cells (Figure 5A), while knockdown of β-catenin increased miR-139-5p expression in DKO-1 and SW620 cells (Figure 5B and Supplementary Figure S2A). In addition, activation of Wnt signaling via Wnt3a treatment reduced miR-139-5p levels in DKs-8 and Caco-2 cells (Figure 5C). In contrast, the β-catenin-CBP inhibitor ICG-001 increased miR-139-5p levels in DKO-1 and SW620 cells (Figure 5D). These results indicate that Wnt/β-catenin signaling inhibits miR-139-5p expression in CRC cells.

TCF3 and TCF4 are correlated with β-catenin and form β-catenin/TCF3 and β-catenin/TCF4 complexes that contribute to CRC progression 34. We thus next determined whether TCF3 or TCF4 is responsible for Wnt/β-catenin signaling-mediated miR-139-5p repression. We found that ectopic expression of TCF4, but not TCF3, suppressed miR-139-5p expression (Figure 5E). Knockdown of TCF4 rescued miR-139-5p expression after Wnt3a treatment (Figure 5F and Supplementary Figure S2B). We further analyzed the promoter region of miR-139-5p with the JASPAR database 35 and identified three putative TCF4 binding sites (Figure 5G). Sequential deletions and mutations of these binding sites revealed that binding sites 1 and 2 are the major sites for TCF4-mediated repression (Figure 5G and 5H, Supplementary Figure S2C). ChIP assays also confirmed that TCF4 binds directly to binding sites 1 and 2 in the promoter of *MIR139* (Figure 5I). These results indicate that miR-139-5p is transcriptionally repressed by Wnt/β-catenin signaling in KRAS-mutant CRC cells.

### Expression patterns of miR-139-5p and its targets in human CRC tissues

**Figure 6:**
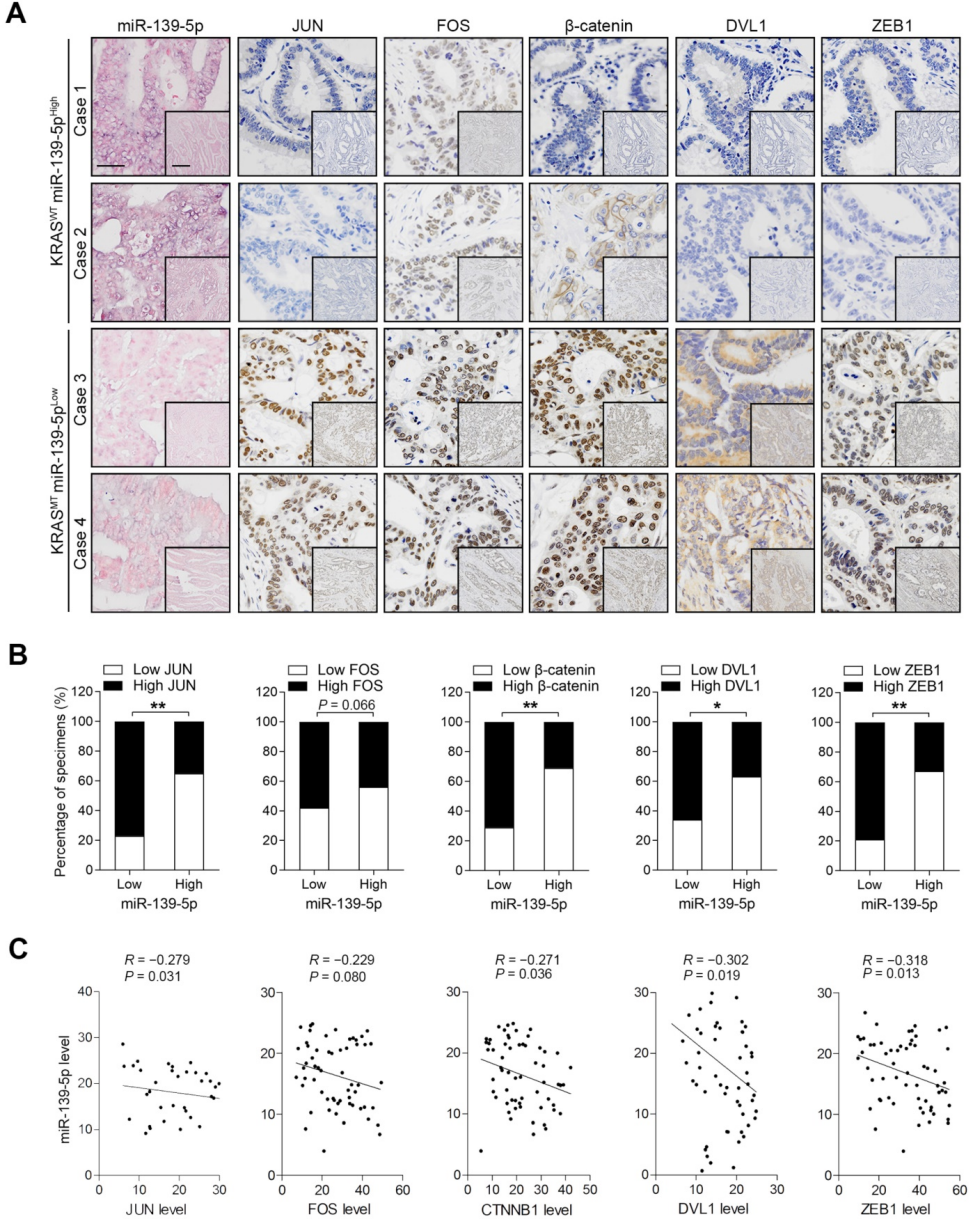
** Expression patterns of miR-139-5p and its targets in human CRC tissues. (A)** ISH of miR-139-5p and IHC of JUN, FOS, CTNNB1, DVL1 and ZEB1 expression in WT (KRAS^WT^) and KRAS-mutant (KRAS^MT^) CRC tissues from the XHDD cohort. Bars: 100 µm (main); 500 µm (inset). **(B)** Associations between miR-139-5p expression and JUN, FOS, CTNNB1, DVL1 and ZEB1 expression in the XHDD cohort CRC tissues. *P<0.05; **P<0.01. **(C)** Correlations between the mRNA expression levels of miR-139-5p and those of JUN, FOS, CTNNB1, DVL1 and ZEB1 in XHDD cohort CRC tissues.

To validate the correlations between miR-139-5p and its targets, we examined the expression of these molecules in the XHDD cohort of 60 CRC patients with and without KRAS mutation (Supplementary Table 1). ISH and IHC revealed that JUN, FOS, DVL1, CTNNB1 and ZEB1 levels were higher in most KRAS^-^mutant samples in which miR-139-5p expression was reduced or lost than in WT KRAS samples in which miR-139-5p was expressed at relatively high levels (Figure 6A). Spearman correlation analysis revealed that the levels of JUN, DVL1, CTNNB1 and ZEB1 were negatively correlated with those of miR-139-5p, but the correlation between FOS and miR-139-5p did not reach statistical significance (Figure 6B). Similar correlations were observed by qRT-PCR analyses using CRC tissues (Figure 6C). These clinical observations further indicate that miR-139-5p suppresses multiple targets in CRC.

## Discussion

Accumulating studies have revealed that driver gene mutations have comprehensive effects on miRNA expression 12, 36, 37. KRAS mutation is one of the most common genetic changes in human cancers 4. Increasing evidence has indicated that KRAS mutations alter miRNA expression and contribute to tumor development and progression. In non-small cell lung cancers, KRAS mutation induces the expression of miR-29b, which represses TET1 expression and thereby transcriptionally silences multiple tumor suppressors 38. In pancreatic cancer, KRAS mutation represses miR-489 through NF-κB/YY1 signaling, thus inhibiting cell migration and metastasis by targeting ADAM9 and MMP7 39. However, in KRAS-mutant CRC, whether miRNAs contribute to KRAS-driven aggressiveness has remained largely unexplored. In the present study, we performed small RNA-Seq using DLD-1 isogenic cell lines to identify KRAS mutation-regulated miRNA signatures in CRC. We ultimately identified a group of KRAS mutation-responsive miRNAs. Among these miRNAs, miR-139-5p was validated to be downregulated in a panel of KRAS-mutant CRC cell lines compared to their WT counterparts. Moreover, we analyzed the expression patterns of miR-139-5p in three different cohorts of CRC patients (the TCGA, XHDD and TMA cohorts) and found similar trends: the levels of miR-139-5p were lower in KRAS-mutant tissues than in WT tissues. These results suggest that miR-139-5p might be a novel effector associated with KRAS mutation in CRC.

miR-139-5p has been previously reported to be downregulated in various types of cancer and to function mainly as a tumor suppressor 16-18, 21, 40. In liver cancer, downregulation of miR-139-5p contributes to cell proliferation, migration and invasion via targeting of ETS1 40. In breast cancer, overexpression of miR-139-5p alleviates radiotherapy resistance by suppressing multiple genes related to DNA repair and ROS defense 16. However, miR-139-5p levels in blood have been reported to be higher in prostate cancer patients than in healthy controls 41. Notably, some previous studies have indicated that miR-139-5p is downregulated in CRC tissues 42, but others have reported that miR-139-5p is upregulated in metastatic CRC 43, 44. Given these conflicting findings, the ambiguous roles of miR-139-5p in CRC require further exploration. In the present study, we obtained several lines of evidence that miR-139-5p functions as a potent tumor suppressor in CRC. First, *in vitro* gain- and loss-of-function experiments showed that miR-139-5p inhibited CRC cell proliferation by inducing apoptosis and cell cycle arrest and repressing cell migration and invasion. Second, *in vivo* tumorigenesis and metastasis assays demonstrated that miR-139-5p suppressed tumorigenicity and hepatic and pulmonary metastasis in mice. Notably, ectopic expression of miR-139-5p increased 5-FU responsiveness in KRAS-mutant CRC cells, suggesting that miR-139-5p could be developed as a therapeutic agent for chemosensitivity enhancement. Third, we found that multiple genes and signaling pathways associated with the cell cycle, adherens junctions and DNA repair cellular processes were influenced by miR-139-5p. These findings partially explain how miR-139-5p functions as a potent tumor suppressor and suggest that it might be a potential prognostic biomarker and therapeutic target for advanced CRC.

TFs can activate or suppress miRNAs to fine-tune certain signaling pathways 45. For instance, HNF4α binds to the miR-124 promoter and activates its expression, after which miR-124 targets IL6R and modulates the IL6R/STAT3 pathway during hepatocellular transformation 46. c-Myc has been shown to transactivate the miR-17-92 cluster, which negatively regulates the expression of E2F1 and controls proliferative signaling in lymphoma cells 47, 48. In the present study, we demonstrated that Wnt/β-catenin signaling suppressed miR-139-5p transcription in a TCF4-dependent manner in KRAS-mutant CRC cells, adding to the body of knowledge regarding miR-139-5p transcriptional regulation. Given that hyperactivation of Wnt/beta-catenin signaling pathway is a critical step in colorectal tumorigenesis, the reciprocal regulation between miRNAs and Wnt signaling represents an important regulatory pattern that orchestrates gene expression in CRC 45, 49. We found that the Wnt signaling regulators CTNNB1 and DVL1 are direct targets of miR-139-5p and therefore contribute to a double-negative feedback loop between Wnt/β-catenin signaling and miR-139-5p. These results provide new insights into KRAS mutation-driven malignancies: miR-139-5p is continuously repressed by Wnt/β-catenin signaling activation and by the reciprocal feedback loop in KRAS-mutant cells. This cellular context-dependent regulatory mechanism may enhance crosstalk among signaling pathways and participate in an elaborate network supporting cancer progression.

## Conclusions

In summary, we found that miR-139-5p is significantly downregulated in KRAS-mutant CRC cells and tissues. miR-139-5p inhibits CRC cell proliferation and metastasis by targeting multiple modulators of the Ras and Wnt signaling pathways and EMT. Transcription of miR-139-5p is suppressed by Wnt/β-catenin signaling in mutant CRC cells. These findings reveal a novel mechanism of miRNA dysregulation and provide promising prognostic biomarkers and therapeutic targets for advanced CRC.

## Figures and Tables

**Table 1:** Correlations between miR-139-5p expression and clinicopathological characteristics in human CRC tissues

Clinicopathological variable		miR-139-5p expression		*P* value
		Low (n=61)	High (n=39)	
Age^1^	≤66.3	41	26	0.473
	>66.3	20	13	
Gender	Female	26	20	0.397
	Male	35	19	
Tumor size	≤5 cm	20	21	0.038
	>5 cm	41	18	
Tumor differentiation	Well or moderate	29	27	0.033
	Poor	32	12	
Tumor invasion	T1	12	12	0.018
	T2	10	10	
	T3	15	13	
	T4	24	4	
Lymph node metastasis	Absent	22	23	0.025
	Present	39	16	
Distant metastasis	Absent	40	33	0.036
	Present	21	6	
AJCC^2^ stage	I	10	13	0.043
	II	12	10	
	III	17	11	
	IV	22	5	

1. Mean age at operation. 2. AJCC: American Joint Committee on Cancer.

**Table 2:** Univariate and multivariate analyses of factors associated with disease-free survival in CRC^1^ patients

Variable	HR^2^	95% CI^3^	*P* value
Univariate analysis			
Age (≤66.3 vs >66.3)	0.998	0.985-1.011	0.772
Sex (female vs male)	1.212	0.916-1.604	0.179
Tumor size (≤5 vs >5 cm)	0.827	0.621-1.101	0.193
Tumor differentiation (well/moderate vs poor)	0.186	0.139-0.249	<0.001
Tumor invasion (T1-T3 vs T4)	0.357	0.265-0.480	<0.001
Lymph node metastasis (absent vs present)	0.136	0.098-0.189	<0.001
Distant metastasis (absent vs present)	0.113	0.081-0.157	<0.001
AJCC^4^ stage (I-II vs III-Ⅳ)	0.133	0.095-0.186	<0.001
miR-139-5p expression (high vs low)	0.250	0.187-0.333	<0.001
Multivariate analysis			
Tumor differentiation (well/moderate vs poor)	0.809	0.544-1.202	0.294
Tumor invasion (T1-T3 vs T4)	0.669	0.218-2.058	0.484
Lymph node metastasis (absent vs present)	0.336	0.114-0.984	0.047
Distant metastasis (absent vs present)	0.485	0.314-0.749	0.001
AJCC stage (I-II vs III-Ⅳ)	0.489	0.344-0.694	<0.001
miR-139-5p expression (high vs low)	0.415	0.300-0.576	<0.001

1. CRC: Colorectal cancer. 2. HR: Hazard ratio. 3. CI: Confidence interval. 4. AJCC: American Joint Committee on Cancer.

## Abbreviations

CRC: colorectal cancer
EMT: epithelial-mesenchymal transition
EGFR: epidermal growth factor receptor
APC: adenomatous polyposis coli
ChIP: chromatin immunoprecipitation
ISH: *in situ* hybridization
IHC: immunohistochemistry
TUNEL: terminal deoxynucleotidyl transferase dUTP nick-end labeling
TCGA: The Cancer Genome Atlas
XHDD: Xijing Hospital of Digestive Diseases
TMA: tissue microarray
TCF/LEF: T cell factor/lymphoid enhancer factor
GSEA: gene set enrichment analysis
KEGG: Kyoto Encyclopedia of Genes and Genomes
GC: gastric cancer
HNF4α: hepatocyte nuclear factor 4 alpha
