# Supplementary material for: KRAS Mutation-Responsive miR-139-5p inhibits Colorectal Cancer Progression and is repressed by Wnt Signaling
Source: Theranostics. 2020 Jun 5;10(16):7335–50. doi: 10.7150/thno.45971 (PMC7330859; doi:10.7150/thno.45971)
Supplement: Supplementary file 1 — Supplementary figures and tables. [file thnov10p7335s1.pdf]

Figure S1

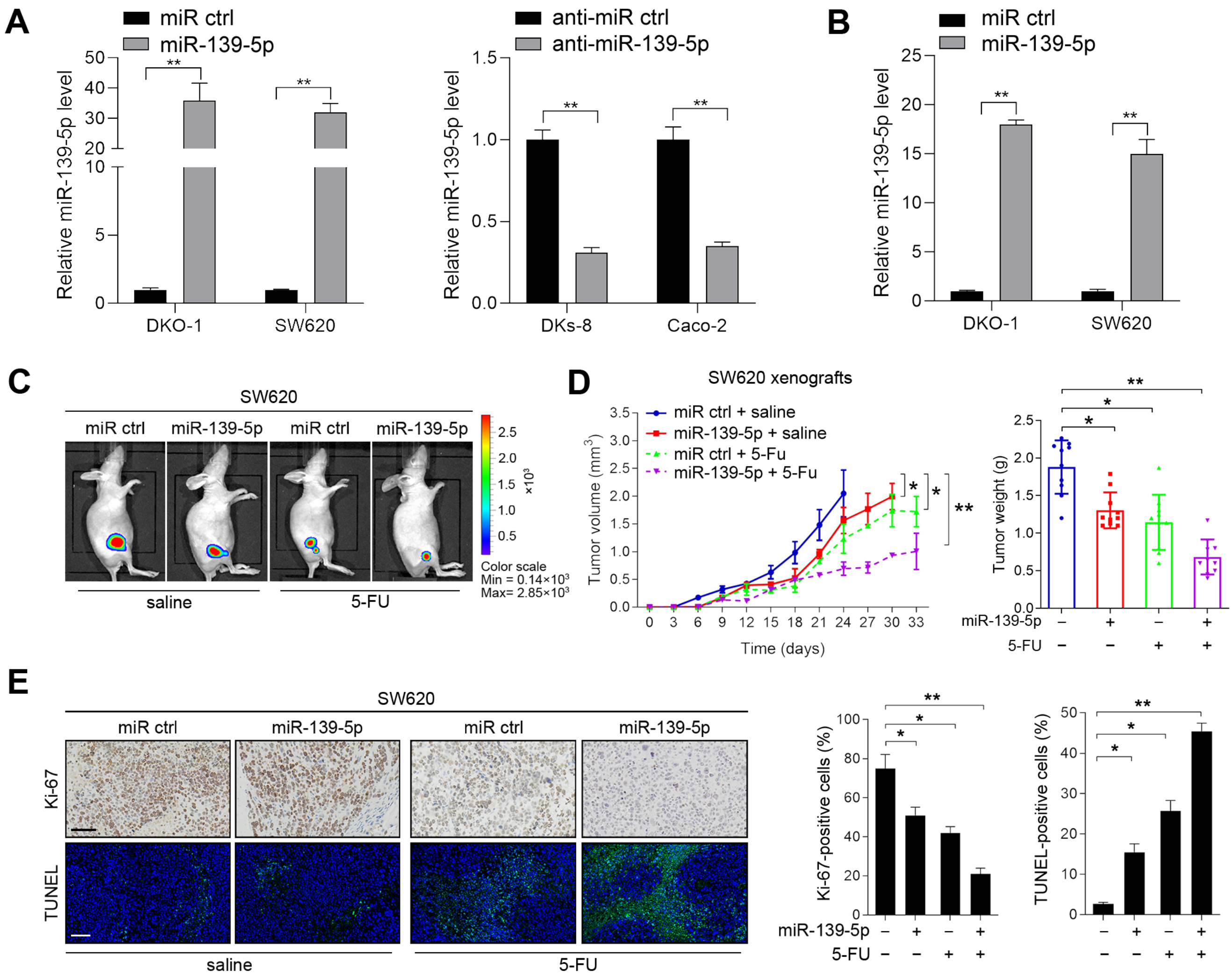
A
miR ctrl
miR-139-5p
Relative miR-139-5p level
DKO-1
SW620
anti-miR ctrl
anti-miR-139-5p
DKs-8
Caco-2
B
miR ctrl
miR-139-5p
Relative miR-139-5p level
DKO-1
SW620
C
SW620
miR ctrl
miR-139-5p
miR ctrl
miR-139-5p
saline
5-FU
Color scale
Min = 0.14×10³
Max= 2.85×10³
D
SW620 xenografts
miR ctrl + saline
miR-139-5p + saline
miR ctrl + 5-Fu
miR-139-5p + 5-Fu
Tumor volume (mm³)
Time (days)
Tumor weight (g)
miR-139-5p
5-FU
E
SW620
miR ctrl
miR-139-5p
miR ctrl
miR-139-5p
Ki-67
TUNEL
saline
5-FU
Ki-67-positive cells (%)
TUNEL-positive cells (%)
miR-139-5p
5-FU

Figure S2

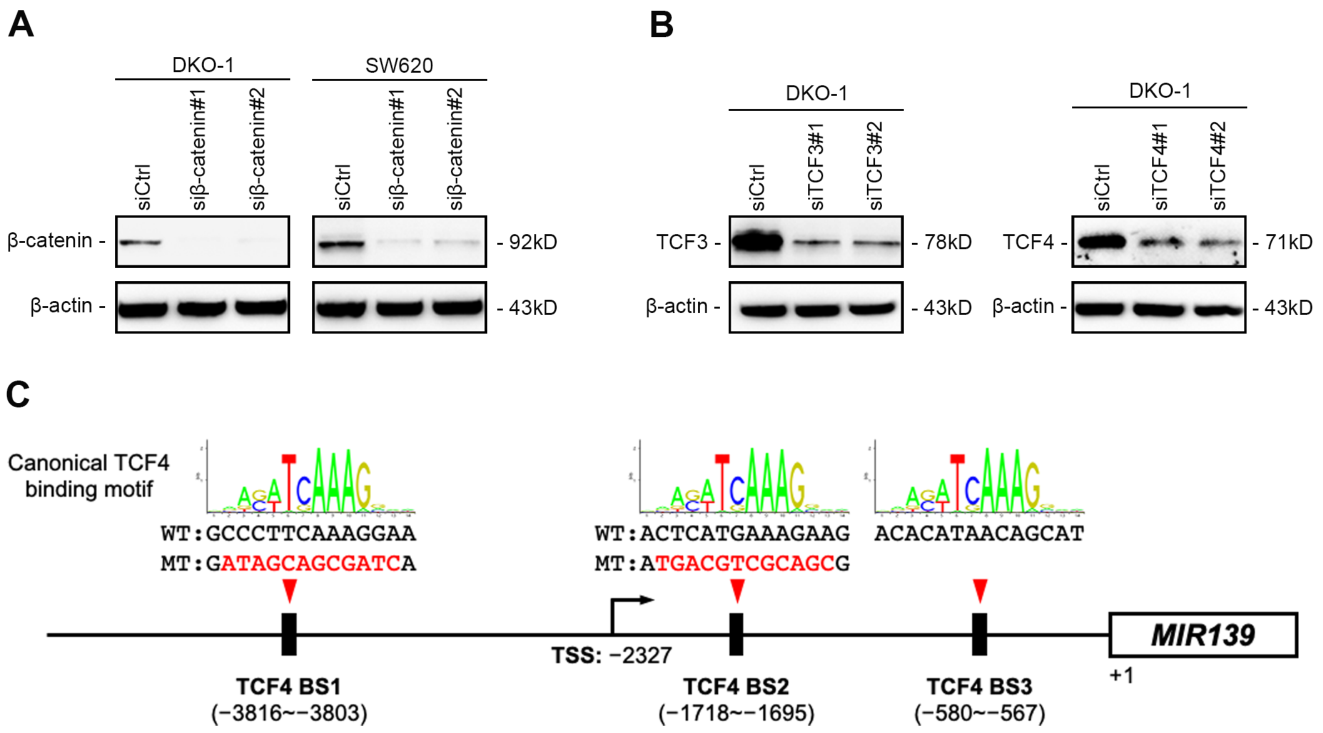

A
DKO-1
SW620
siCtrl
siβ-catenin#1
siβ-catenin#2
β-catenin -
β-actin -
- 92kD
- 43kD
B
DKO-1
siCtrl
siTCF3#1
siTCF3#2
siTCF4#1
siTCF4#2
TCF3 -
TCF4 -
- 78kD
- 71kD
C
Canonical TCF4 binding motif
WT:GCCCTTCAAAGGAA
MT:GATAGCAGCGATCA
WT:ACTCATGAAAGAAG
MT:ATGACGTCGCAGCG
ACACATAACAGCAT
TSS: −2327
MIR139
+1
TCF4 BS1
(−3816~−3803)
TCF4 BS2
(−1718~−1695)
TCF4 BS3
(−580~−567)

Supplementary Figure Legends

Figure S1. (A) The indicated DKO-1 and SW620 cells or DKs-8 and Caco-2 cells were transfected with a miR-139-5p agomir or antagomir, respectively, using Lipofectamine 2000 according to the manufacturer's instructions. The transfection efficiencies of the miR-139-5p agomir and antagomir were validated by qRT-PCR. **(B)** To generate stable cell lines, DKO-1 and SW620 cells were infected with lentiviruses at a multiplicity of infection of 100:1. Infection efficiency was confirmed by qRT-PCR 72 h after infection. **(C)** The indicated cells were injected subcutaneously into nude mice (n = 10). After the tumor size reached approximately 100 mm^3, the mice received 5-FU treatment (8 mg/kg/d, i.p. injection). Representative fluorescent images of GFP signals captured from subcutaneous tumors are shown. **(D)** Left, growth curves of tumors in nude mice injected with the indicated cells. Right, calculated weights and volumes of tumors isolated on day 30 after treatment. The results were analyzed by one-way ANOVA followed by Dunnett's test compared with the miR ctrl group. **(E)** Left, representative images of tumor samples that were subjected to IHC for Ki-67 and TUNEL staining. Right, percentages of Ki-67- and TUNEL-positive cells. Bars: 200 μm. The data are presented as the means ± SDs. *, $P < 0.05$; **, $P < 0.01$, n.s., not significant.

Figure S2. (A) Transfection with β-catenin siRNAs significantly decreased β-catenin protein levels in DKO-1 and SW620 cells. siβ-catenin#1 was used in Fig. 5B. **(B)** Transfection with TCF3 and TCF4 siRNAs significantly decreased TCF3 and TCF4 protein levels in DKO-1 cells. siTCF3#1 and siTCF4#2 were used in Fig. 5F. **(C)** Schematic of the predicted TCF4 binding sites in the promoter region of the *MIR139* sequence. The indicated TCF4 binding sites are mapped to their respective locations in *MIR139* and are numbered relative to the *MIR139* sequence (+1). The sequence of each site and the bases mutated in the mutant promoters are shown.

Supplementary Table 1. Clinic-pathological characteristics of CRC patients with paired adjacent non-tumor tissues.

No[1]	Age	Gender	TNM stage	KRAS status[2]
1	60	Female	T3N1aM0	WT
2	65	Female	T2N0M0	WT
3	59	Female	T3N0M0	WT
4	72	Male	T3N0M0	WT
5	72	Male	T3N0M0	WT
6	59	Female	T4bN0M0	WT
7	72	Male	T2N1bM0	WT
8	69	Male	T3N0M0	WT
9	54	Male	T3N1aM0	WT
10	53	Male	T3N1aM0	WT
11	59	Female	T2N0M0	WT
12	47	Male	T2N0M0	WT
13	78	Male	T3N0M0	WT
14	48	Male	T3N0M0	WT
15	54	Male	T3N1aM0	WT
16	54	Male	T3N0M0	WT
17	63	Male	T2N0M0	WT
18	47	Female	T2N0M0	WT
19	46	Male	T3N0M0	WT
20	64	Female	T3N0M0	WT
21	70	Male	T4aN2bM0	WT
22	74	Male	T3N0M0	WT
23	72	Male	T2N0M0	WT
24	67	Male	T2N0M0	WT
25	59	Male	T3N0M0	WT
26	53	Female	T3N0M0	WT
27	53	Male	T3N0M0	WT
28	84	Female	T3N0M0	WT
29	82	Female	T3N0M0	WT
30	67	Male	T3N0M0	WT
31	62	Female	T3N1aM0	Exon2 G13D
32	60	Female	T3N1bM0	Exon2 G13C
33	84	Male	T2N0M0	Exon2 G12/G13C
34	69	Male	T3N0M0	Exon2 G13D
35	46	Female	T4aN2bM0	Exon2 G13D
36	73	Female	T4aN2aM0	Exon2 G13D

Case No[1]	Age	Gender	TNM stage	KRAS status
37	50	Female	T2N0M0	Exon2 G13D
38	57	Male	T3N0M0	Exon2 G12X/G13C
39	75	Male	T3N2aM0	Exon2 G12S/G13D
40	73	Male	T3N1cM0	Exon2 G12S/G12D
41	70	Male	T3N0M0	Exon2 G12S/G12D
42	80	Male	T3N0M0	Exon2 G12X/G13C
43	70	Male	T4bN1aM1	Exon2 G12S/G12D
44	52	Female	T3N0M0	Exon2 G12S/G12D
45	62	Male	T2N0M0	Exon2 G12X/G13C
46	69	Male	T3N1cM0	Exon2 G12X/G13C
47	58	Female	TisN0M0	Exon2 G12X/G13C
48	59	Male	T2N0M0	Exon2 G12X/G13C
49	55	Female	T3N1bM0	Exon2 G12X/G13C
50	76	Female	T4bN0M0	Exon2 G12S/G12D
51	71	Male	T3N1cM0	Exon2 G12S/G12D
52	71	Female	T4aN0M0	Exon2 G12S/G12D
53	73	Female	T3N0M0	Exon2 G12X/G13C
54	61	Male	T4aN1bM0	Exon2 G13D
55	59	Male	T3N1cM0	Exon2 G13D
56	59	Female	T3N1aM0	Exon2 G13D
57	75	Male	T3N1bM0	Exon2 G13D
58	64	Male	T3N1cM0	Exon2 G12X/G13C
59	28	Male	T2N1M0	Exon2 G12X/G13C
60	72	Female	T3N0M0	Exon2 G12X/G13C

1. Cases 1, 2, 3, and 4 in Fig. 6A denote Subjects No. 2, 24, 32, and 54 in this table.

2. WT, wild-type.

Supplementary Table 2. Schema of miR-139-5p binding sites in predicted target 3' UTR sequences of human genes Gene.

<table>
<tr><th>Gene</th><th>Target Site</th><th>3' UTR Position</th></tr>
<tr><td>PRKCA</td><td>5' ...AUGACAGGCCUGGAGCUGUAGAA...
|||| ||||||
3' UGACCUCUGUGCACGU---GACAUCU</td><td>1151-1157</td></tr>
<tr><td>PRKCA</td><td>5' ...GUGUCCAGUUUAAUUCUGUAGAA...
||||||
3' UGACCUCUGUGCACGUGACAUCU</td><td>234-240</td></tr>
<tr><td>NFAT5</td><td>5' ...AGAAUAUAUACCUGAACUGUAGU...
|||||||
3' UGACCUCUGUGCACGUGACAUCU</td><td>3004-3010</td></tr>
<tr><td>ZEB1</td><td>5' ...AGUGUAGUGUAUAAUACUGUAGU...
|||||||
3' UGACCUCUGUGCACGUGACAUCU</td><td>1424-1430</td></tr>
<tr><td>ZEB1</td><td>5' ...UUAUACUUGCCUUGGACUGUAGA...
|||||||
3' UGACCUCUGUGCACGUGACAUCU</td><td>1565-1572</td></tr>
<tr><td>RASGRF1</td><td>5' ...GAUAAUUAGUACCACACUGUAGA...
|||||||
3' UGACCUCUGUGCACGUGACAUCU</td><td>887-894</td></tr>
<tr><td>RASGRF1</td><td>5' ...GUUUUUAUUGCAUAUACUGUAGU...
|||||||
3' UGACCUCUGUGCACGUGACAUCU</td><td>932-938</td></tr>
<tr><td>RASGRF1</td><td>5' ...CUUGUUCUUAGAUUCCUGUAGAA...
||||||
3' UGACCUCUGUGCACGUGACAUCU</td><td>138-144</td></tr>
<tr><td>JUN</td><td>5' ...CAAACUGCAAUAGAGACUGUAGA...
|||||||
3' UGACCUCUGUGCACGUGACAUCU</td><td>377-384</td></tr>
<tr><td>JUN</td><td>5' ...UCUUUUCUGCAUCAUCUGUAGAU...
||||||
3' UGACCUCUGUGCACGUGACAUCU</td><td>782-788</td></tr>
<tr><td>DVL1</td><td>5' ...CCCCACGUGUCUGUGCUGUAGAU...
|||||| ||||||
3' UGACCUCUGUGCACGU----GACAUCU</td><td>759-765</td></tr>
<tr><td>FOS</td><td>5' ...UAGCUAUAUCCAUGUACUGUAGU...
|||||||
3' UGACCUCUGUGCACGUGACAUCU</td><td>526-532</td></tr>
<tr><td>FOS</td><td>5' ...UUCCCUAGAGGGUUCCUGUAGAC...
||||||
3' UGACCUCUGUGCACGUGACAUCU</td><td>96-102</td></tr>
<tr><td>CACNA2D1</td><td>5' ...GUUGUUUUUUCUCUUACUGUAGA...
|||||||
3' UGACCUCUGUGCACGUGACAUCU</td><td>942-949</td></tr>
<tr><td>CCND2</td><td>5' ...UAAAGUGCCUUACUGACUGUAGC...
|||||||
3' UGACCUCUGUGCACGUGACAUCU</td><td>4739-4745</td></tr>
</table>

MAPK8	5' ...UGGGCCUCUGGGCUGCUGUAGAU...<br>|||||| 3' UGACCUCUGUGCACGUGACAUCU	125-131
MAPK8	5' ...GUUUACAUUUUCUAUCUGUAGAA...<br>||| |||||| 3' UGACCUCUGUGCACGU--GACAUCU	1475-1481
FZD3	5' ...GAACUGUGUUUUUAAACUGUAGG...<br>||||||| 3' UGACCUCUGUGCACGUGACAUCU	493-499
FZD3	5' ...UCCCAAGUAGCUAAGACUGUAGG...<br>||||||| 3' UGACCUCUGUGCACGUGACAUCU	4107-4113
FZD3	5' ...UGUGGAUCGCUCCUCCUGUAGAU...<br>|||||| 3' UGACCUCUGUGCACGUGACAUCU	9124-9130
RAP1B	5' ...UUCAGGCUUCUGCAGCUGUAGAU...<br>|||||| 3' UGACCUCUGUGCACGUGACAUCU	824-830
CTNNB1	5' ...UGCAUUGUGAUUGGCCUGUAGAG...<br>|||||| 3' UGACCUCUGUGCACGUGACAUCU	349-355
CAMK2D	5' ...UGGUGUUGAAGUAUUACUGUAGA...<br>||||||| 3' UGACCUCUGUGCACGUGACAUCU	1938-1945
PPP2CA	5' ...GCUUUACUCAGCAUGACUGUAGA...<br>||||||| 3' UGACCUCUGUGCACGUGACAUCU	365-372
ROCK1	5' ...AACUUCAGUGCCCUCACUGUAGA...<br>||||||| 3' UGACCUCUGUGCACGUGACAUCU	538-545
TCF4	5' ...UUUGCUAUAAAAUAGACUGUAGU...<br>||||||| 3' UGACCUCUGUGCACGUGACAUCU	3950-3956
ROCK2	5' ...AGAAAACUAAGACAG----ACUGUAGA...<br>||||| ||||||| 3' UGACCUCUGUGCACGUGACAUCU	1156-1163

Supplementary Table 3. Primer sequences used in the study.

Primer name	Primer sequences
**Primers used for Sanger sequencing of KRAS**	
KRAS-exon2 (G12, G13) sense	5'-GTTCTAATATAGTCACATTTTCA-3'
KRAS-exon2 (G12, G13) antisense	5'-TCTATTGTTGGATCATATTCG-3'
KRAS-exon3 (Q61) sense	5'-TCTCCCTTCTCAGGATTC-3'
KRAS-exon3 (Q61) antisense	5'-ATTATTTATGGCAAATACACAAAG-3'
KRAS-exon4 (A146) sense	5'-TTCTAGAACAGTAGACACAAAAC-3'
KRAS-exon4 (A146) antisense	5'-GAGAGAAAAACTGATATATTAAATGAC-3'
KRAS-exon4 (K117) sense	5'-CTTTCCCAGAGAACAAATTAAAAG-3'
KRAS-exon4 (K117) antisense	5'-TCAATAAAAGGAATTCCATAACTTCT-3'
**Primers for TCF4 site-directed construct in miR-139-5p promoter**	
(-5000/-1bp) sense	5'-TATAGGTACCAAGGCCAAAGTCTCTTAACCG-3'
(-3802/-1bp) sense	5'-TATAGGTACCAATAACTCTGGATGTCAGCCTG-3'
(-1694/-1bp) sense	5'-TATAGGTACCGTGATAGTGACAGTGCTCCTC-3'
(-566/-1bp) sense	5'-TATAGGTACCTTGGGAGGAAGTTTGTGGTGTC-3'
Antisense	5'-ATATAAGCTTCTGAGCCAGTCCCAGTGCCTCC-3'
**Primers for TCF4 site-directed mutagenesis in miR-139-5p promoter**	
binding site 1 mutation sense	5'-GGCTTCCTGGGatagcagcgatcAAATAACTCTG-3'
binding site 1 mutation antisense	5'-CAGAGTTATTTgatcgctgctatCCCAGGAAGCC-3'
binding site 2 mutation sense	5'-AGGTGTCCTCAtgacgtcgcagcGGTGATAGTGA-3'
binding site 2 mutation antisense	5'-TCACTATCACCgctgcgacgtcaTGAGGACACCT-3'
**Primers used for ChIP against TCF4 in the miR-139-5p promoter**	
distant region sense	5'-AAAGTCTCTTAACCGTGCAG-3'
distant region antisense	5'-TCATCCGTAAAATGGTCTCT-3'
binding site 1 sense	5'-CTGACATATTTACCCAGCCC-3'
binding site 1 antisense	5'-ACTCTGCCTCATTACACAGG-3'
binding site 2 sense	5'-GATTGGACTACATGAGGCCT-3'
binding site 2 antisense	5'-GAGAGCCTTTGGCAACCTCT-3'
